# Investigating the neural substrates of Antagonistic Externalizing and social-cognitive Theory of Mind: an fMRI examination of functional activity and synchrony

**DOI:** 10.1017/pen.2020.12

**Published:** 2021-01-19

**Authors:** Brandon Weiss, Andrew Jahn, Courtland S. Hyatt, Max M. Owens, Nathan T. Carter, Lawrence H. Sweet, Joshua D. Miller, Brian W. Haas

**Affiliations:** 1 University of Georgia Franklin, College of Arts and Sciences, Psychology, Athens, Georgia; 2 University of Michigan, fMRI Laboratory, Ann Arbor, Michigan; 3 University of Vermont, Burlington, Vermont

**Keywords:** Externalizing, Antagonism, Personality, Theory of Mind, Social cognition

## Abstract

Recently developed quantitative models of psychopathology (i.e., Hierarchical Taxonomy of Psychopathology) identify an Antagonistic Externalizing spectrum that captures the psychological disposition toward criminal and antisocial behavior. The purpose of the present study was to examine relations between Antagonistic psychopathology (and associated Five-Factor model Antagonism/Agreeableness) and neural functioning related to social-cognitive Theory of Mind using a large sample (*N* = 973) collected as part of the Human Connectome Project (Van Essen et al., 2013a). No meaningful relations between Antagonism/Antagonistic Externalizing and Theory of Mind-related neural activity or synchrony were observed (*p* < .005). We conclude by outlining methodological considerations (e.g., validity of social cognition task and low test–retest reliability of functional biomarkers) that may account for these null results, and present recommendations for future research.

A major contemporary challenge for clinical science is to identify neurobiological dimensions underlying psychopathological processes. Reflecting this understanding, the National Institute of Mental Health created the Research Domain Criteria (Cuthbert & Insel, 2013) initiative to explore the genetic, biological, and behavioral underpinnings of psychopathology and investigate social processes relevant to the perception and understanding of others. So far, a small body of research has emerged examining the neurobiological link between personality traits and a category of cognitive functions defined as Theory of Mind (ToM; i.e., mentalizing, empathy, and social cognition). Specifically, the Agreeableness domain of the Five-Factor Model (FFM; Costa & McCrae, 1992) of personality has shown evidence of being connected to ToM performance (e.g., Allen, Rueter, Abram, Brown & DeYoung, 2017; Nettle & Liddle, 2008). This suggests that lower-order ToM cognition, in subserving the ability to reason about the mental states of others, may underlie higher-order patterns of thought, feeling, and behavior relevant to social information processing.

Meanwhile, recent advances in the measurement of personality pathology provide the opportunity to investigate links between personality, psychopathology, and neurocognition with greater precision. Some recent findings indicate that putatively separable personality disorders involving externalizing behaviors (e.g., antisocial personality disorder [APD], narcissistic personality disorder [NPD], psychopathy) are better conceptualized as heterogeneous constructs that hold certain personality dimensions in common, namely (low) FFM Agreeableness and Conscientiousness (O’Connor, 2005; Widiger, Lynam, Miller & Oltmanns, 2012). The Hierarchical Taxonomy of Psychopathology (HiToP; Kotov et al., 2017), a data-driven and integrative model of psychopathology, reflects these dimensions in two Externalizing spectra (Antagonistic Externalizing and Disinhibited Externalizing). Antagonistic Externalizing, which describes tendencies toward engaging in criminal and antisocial acts involving theft, destruction of property, physical aggression toward others, and violations of rules of conduct, is most relevant to the present study in view of its relations to FFM Agreeableness and reference to interpersonal conduct. Focusing on coherent dimensions of personality and psychopathology (versus categorical disorders [e.g., APD]) is thought to be favorable to uncovering links between personality/psychopathology and neurocognition because irrelevant traits (e.g., Disinhibition within APD) are no longer detracting from the signal of relevant ones. A major research initiative is to identify the neurocognitive mechanisms by which these higher-order latent factors impact life outcomes.

## 1.1. ToM, agreeableness, and antagonistic externalizing

ToM has been broadly separated into social-affective (also known as social-perceptual) and social-cognitive perception components (Meinhardt-Injac, Daum, Meinhardt & Persike, 2018). The social-affective component represents the capacity to infer an individual’s affective mental state from external information involving physical expression (e.g., facial cues and nonverbal behaviors), whereas the social-cognitive component represents the capacity to infer mental states from more subtle and complex contextual cues that are not physically apparent. Measures of social-affective and social-cognitive ToM are moderately related (e.g., Fossati, Somma, Krueger, Markon & Borroni, 2017 [*r* = .36]), with some evidence to suggest that the two components rely on distinct neural circuits. The social-affective perception component is believed to be tied to orbitofrontal/medial temporal/amygdala circuits, whereas the social-cognitive perception component is believed to be mediated by the medial prefrontal cortex (particularly dorsally and in the left hemisphere), the temporoparietal junction (TPJ), and precuneus (Allen et al., 2017; Haas, Anderson & Filkowski, 2015; Sabbagh, 2004; Saxe & Kanwisher, 2003; Spunt & Lieberman, 2012; Young, Dodell-Feder & Saxe, 2010). Notably, the latter circuit meaningfully overlaps with the Default Mode Network (DMN; Allen et al., 2017), which shows activation across contexts in which individuals passively generate mental content involving social cognition, memories, and imagination (Spreng & Andrews-Hanna, 2015). In this study, we focused on brain regions associated with social-cognitive ToM (e.g., Ewing, Zeigler-Hill & Vonk, 2016; Nettle & Liddle, 2008), and “ToM” will heretofore refer to social-cognitive ToM, specifically.

The link between Antagonistic Externalizing and ToM is based on (a) a close connection between low FFM Agreeableness (or FFM Antagonism at the opposite pole) and the Antagonistic Externalizing dimension of HiToP and (b) the theory that low FFM Agreeableness may represent a higher-order manifestation of deficits in social information processing, which may impede core traits related to Agreeableness including compassion and empathy (e.g., DeYoung & Gray, 2009). Specifically, social-cognitive deficits in inferring the mental states of others may underlie Antagonistic Externalizing through impairing individuals’ capacity for insight into others’ sensibilities, preferences, and boundaries using subtle, contextual cues that are not physically apparent. Lack of knowledge of these sensibilities may lead to reduced wherewithal or concern for supporting others’ well-being, cooperating in the pursuit of collective goals, or appreciating the value of rules. ToM deficits may also affect behavior in more indirect ways such as through impairing insight into the untoward elements of one’s own social behavior (via processing social feedback) and limiting prosocial aspects of socialization.

Empirically, the proposed connection between Antagonistic Externalizing and ToM rests primarily on links between FFM Agreeableness and ToM performance. Four studies point to a meaningful relation between FFM Agreeableness and ToM, as assessed by comprehension tasks (i.e., assessing insight into a characters’ use of deception, faux pas, persuasion, false belief, etc., within verbal scenarios) and a novel reaction-time-based emotion attribution task (e.g., Allen et al., 2017; Ewing et al., 2016; Haas, Ishak, Denison, Anderson & Filkowski, 2015; Nettle & Liddle, 2008). Perhaps the most informative study examined aspects of FFM Agreeableness in a moderately sized sample while controlling for memory and IQ (Allen et al., 2017). The authors found differential relations between aspects of Agreeableness and ToM, with Compassion (*β* = .11) and Non-aggression (*β* = .21) factors showing positive relations, and an Honesty factor (*β* = −.25) showing a negative relation.

Direct examinations of Antagonistic Externalizing in relation to ToM have not yet been conducted. Although evidence from examinations of categorical disorders (e.g., NPD and psychopathy) is fairly plentiful, results from this research domain have been strongly mixed (e.g., Blair et al., 1996; Drayton, Santos & Baskin-Sommers, 2018; Fonagy, Target, Steele & Steele, 1998; Nentjes, Bernstein, Arntz, Slaats & Hannemann, 2015; Pellecchia et al., 2015; Vonk et al., 2015). Inconsistency in these findings is likely to be a natural effect of poor measurement accompanying structural heterogeneity in constructs such as NPD, psychopathy, and APD (Lynam, Gaughan, Miller, Mullins-Sweatt & Widiger, 2011; Saulsman & Page, 2004).

Evidence for neural links between Agreeableness and ToM is similarly limited. Only one moderately sized study has directly examined relations between FFM Agreeableness and DMN processing (Allen et al., 2017). Using functional connectivity analyses of resting-state functional magnetic resonance imaging (fMRI), the study found generally null results, though a negative relation between an Honesty factor of Agreeableness and DMN connectivity (*p* < .05) was found that did not survive statistical correction. However, it bears noting that participants were not specifically engaging in ToM but rather were engaged in undirected cognition that may or may not have included engagement of ToM. Given that the DMN serves a variety of functions, measuring its activity at rest likely captures a variety of processes of which not all are likely to be related to Agreeableness. In sum, a small body of consistent findings demonstrate a link between FFM Agreeableness and ToM using behavioral measures, but relations between FFM Agreeableness and ToM-related neural processing are very limited, and the role of ToM in Antagonistic Externalizing has not been directly or validly tested.

## 1.2. Present study

The purpose of the present study was to build upon extant research by using a task-based functional neuroimaging methodology to investigate relations between (low) FFM Agreeableness (heretofore referred to as FFM Antagonism), Antagonistic Externalizing symptoms, and social-cognitive ToM. Although FFM Antagonism and Antagonistic Externalizing are highly related, we examine both separately in acknowledgement that they may capture different ranges of the trait spectrum of Antagonism. To investigate, we used a large sample from existing data collected as part of the Human Connectome Project (HCP; Van Essen et al., 2013a). To measure ToM, we used a social cognition task developed by Castelli and colleagues (2000) and Wheatley and colleagues (2007) to elicit ToM social cognition and associated neural function. To operationalize ToM neural response, we contrasted the activation in the brain’s social cognition system during ToM sequences and Random control sequences, consistent with prior literature (Castelli, Happe, Frith & Frith, 2000; Castelli, Frith, Happe & Frith, 2002; Wheatley, Milleville & Martin, 2007).

Our primary hypothesis was that FFM Antagonism and Antagonistic Externalizing psychopathology would bear negative relations to neural activity in and synchrony among brain regions implicated in ToM. Only one study has directly tested relations between Agreeableness and neural synchrony in regions implicated in ToM, showing marginally significant results (Allen et al., 2017). The present study builds upon Allen and colleagues’ (2017) work by utilizing a social-cognitive task to elicit ToM-related brain response. In addition, we also carried out a series of exploratory analyses. Relations between other personality domains (i.e., Neuroticism, Extraversion, Openness, Disinhibition [reversed Conscientiousness]) and internalizing psychopathology, on one hand, and neural activity and synchrony, on the other, during the ToM paradigm was examined. Multivariate analyses were also conducted in which relations with Antagonism were examined while controlling for other FFM domains.^1^

## 2. Method

### 2.1. Participants

Self-reported personality, psychopathology, and fMRI data were collected from 1,206 subjects who participated in the HCP (Van Essen et al., 2013a, 2013b). Informed consent was obtained for all participants. Participants were community-dwelling healthy adults between 22 and 35 years old with no significant history of psychiatric disorder, substance abuse, neurological disorder or damage, cardiovascular disease, or Mendelian genetic disease. Full fMRI social cognition task data were available for 1,054 participants. Fifty-six participants were excluded from analyses who failed to accurately report social stimuli following ToM sequences 62% of the time or greater (62% corresponded to one standard deviation greater (~12%) than chance (i.e., 50%). Additional participants were excluded due to missing validity check data (one participant), missing personality or psychopathology data (four participants), and incorrect processing of right-left runs (20 participants). Although the latter group of participants possessed correct left-right runs, only participants containing both runs were included to mitigate phase encoding bias. This yielded a final sample of 973 participants. Demographic information for this sample is found in Table 1.

**Table 1.** Demographic information on the current sample (*N* = 973)

	Percentage/mean (SD)
Sex	
Male	46.13%
Female	53.87%
Years of age	29.73 (3.74)
Race	
White or Caucasian	76.77%
African American	12.95%
Asian American	6.27%
Native American	.10%
More than one race	2.36%
Not sure or unknown	1.54%
Ethnicity	
Hispanic or Latino	8.74%
Not Hispanic or Latino	90.03%
Not sure or unknown	1.23%

### 2.2. Materials and procedures

#### 2.2.1. Personality

Traits from the Five Factor Model (FFM) of personality were measured using the NEO Five Factor Inventory (NEO-FFI; Costa & McCrae, 1992), which is a 60-item measure that indexes characteristic patterns of thoughts, emotions, and behaviors. Similar to our strategy of reversing NEO-FFI Agreeableness and referring to it by its low pole, Antagonism, NEO-FFI Conscientiousness scores were reversed and referred to as Disinhibition in line with the present study’s focus on psychopathological aspects of personality. Reliability estimates of internal consistency ranged from .75 (Openness) to .85 (Neuroticism).

#### 2.2.2. Psychopathology

Psychopathology was measured using the Achenbach Adult Self-Report (ASR) scale of adaptive functioning, a 123-item measure of adaptive functioning for adults that includes items on emotional, behavioral, and social problems (Achenbach & Rescorla, 2003). Responses produce both externalizing and internalizing psychopathology composites which were used in present analyses. The Externalizing Psychopathology composite is comprised of Aggression, Rule-Breaking, and Intrusive Behavior subscales, whereas the Internalizing Psychopathology composite is comprised of Anxiety/Depression, Withdrawnness, and Somatic Complaints subscales. Although the externalizing psychopathology composite is not a pure measure of HiToP Antagonistic Externalizing, it is likely to index Antagonistic Externalizing more strongly than Disinhibited Externalizing in view of its stronger relations with FFM Antagonism (*r* = .47) versus Disinhibition (*r* = .31; Table 3), and the presence of exclusively weak item loadings from ASR Externalizing onto the Conscientiousness factor is in Table S7. Therefore, we use ASR Externalizing to refer to Antagonistic Externalizing in the Results section. A reliability estimate of internal consistency was computed for each index: Internalizing (*α* = .88) and Externalizing (*α* = .86).

#### 2.2.3. ToM task

**Figure 1.**
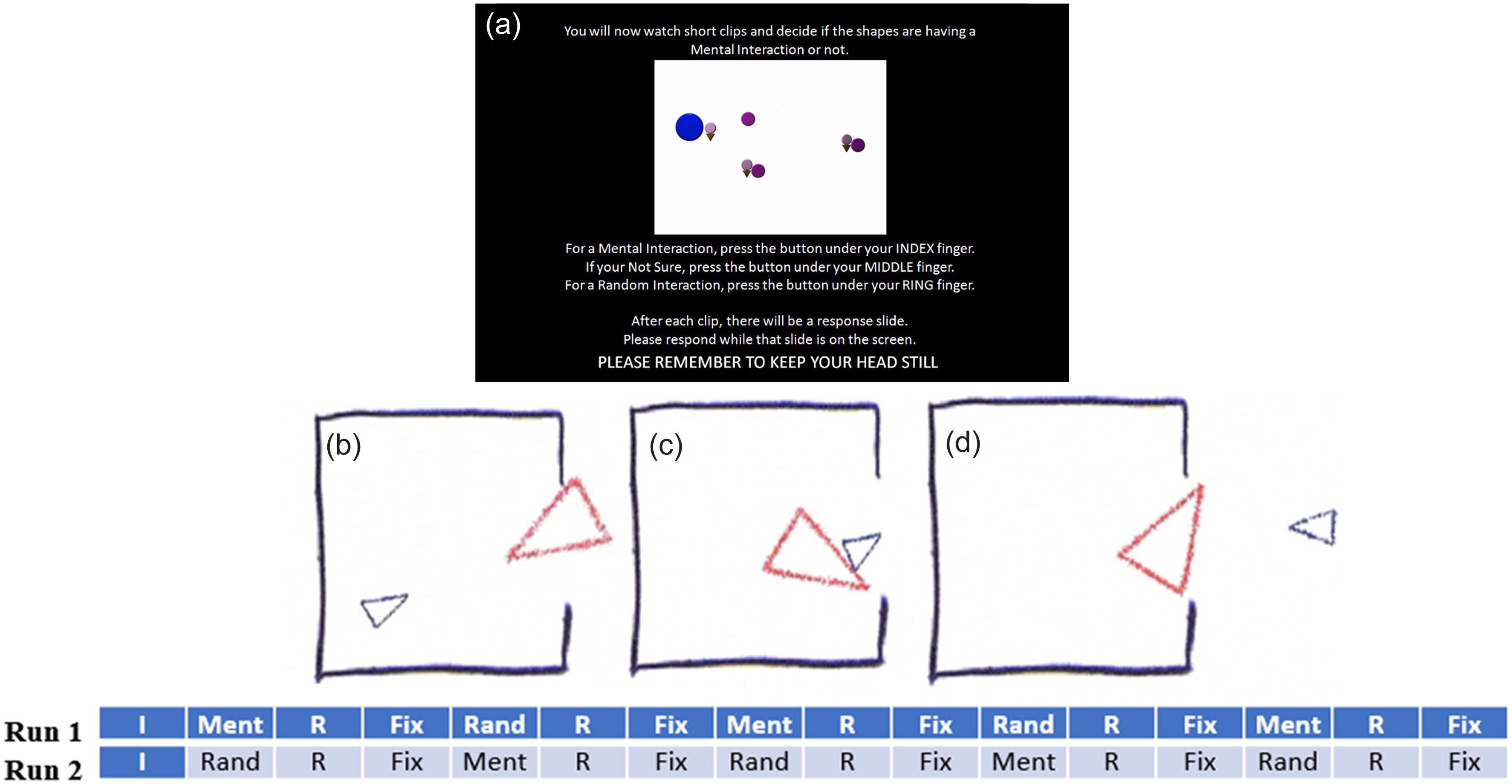
Social cognition task design. Note. I = Instructions (5 s); Ment = Mentalizing Scene (20 s); Rand = Random Scene (20 s); R = Response (3 s); Fix = Fixation/Rest (15 s); Panel A = Task Instructions; Panel B-D = Example Mentalizing Scene.

An fMRI social cognition task was used to elicit ToM. In this task, fMRI imaging was conducted during a video clip task in which subjects observed objects (squares, circles, and triangles) either interacting with each other in an animate manner (ToM sequence) or moving randomly (Random sequence). Developed by Castelli and colleagues (2000) and Wheatley and colleagues (2007), the video clips in the experimental task condition were designed to stimulate social-cognitive ToM and have shown reliable associations with activation in ToM-related brain regions (Castelli et al., 2000, 2002; Wheatley et al., 2007; White et al., 2011). Each subject received two task/imaging runs in which five video blocks were administered (two ToM and three Random blocks in an imaging run acquired from right to left; three ToM and two Random blocks in an imaging run acquired from left to right). Of note, Castelli and colleagues’ (2000) video clips were shortened from 40 sto 20 s to accommodate integrated task paradigm. A pilot study confirmed that the shorter video clips elicited similar responses to the longer video clips (Barch et al., 2013). The task’s design is presented in Figure 1.

#### 2.2.4. Functional magnetic resonance imaging protocol

fMRI data were collected during the social cognition task using a 32-channel head coil on a 3 T Siemens Skyra (Barch et al., 2013). A multi-band acceleration factor of 8 was used. Two task fMRI runs lasting 3:12 were completed with a repetition time (TR) = 720 ms, echo time (TE) = 33.1 ms, flip angle = 52 degrees, field of view (FOV) = 208 × 180 mm, 72.2-mm-thick sagittal slices, and 2.0 mm isotropic voxels. Data were downloaded from the HCP database having been preprocessed using the minimal preprocessing pipeline (Glasser et al., 2013). This pipeline includes gradient unwarping, field-map-based echo-planar imaging (EPI) distortion correction, motion correction, registration of EPI to the structural scan and into MNI152 space, and grand-mean intensity normalization.

#### 2.2.5. Operationalization of ToM fMRI response

All fMRI data processing and analysis were conducted using Statistical Parametric Mapping-12 software (SPM12; Wellcome Department of Imaging Neuroscience, London, UK) and implemented through Matlab R2013a (www.mathworks.com). Spatial smoothing was done to minimally preprocessed data using a 5 mm full width half maximum Gaussian filter. A whole-brain voxel-wise analysis was performed by creating a general linear model with the following regressors: (a) A 20-s boxcar regressor convolved with each condition (ToM sequences and Random sequences); (b) six dimensions of motion (x, y, z, roll, pitch, yaw); and (c) linear, quadratic, and cubic trends. Consistent with prior work using this social cognition task (e.g., Castelli et al., 2002), parameter estimates for the blood-oxygen-level-dependent (BOLD) response during ToM sequences (i.e., when shapes displayed social animacy) was contrasted with response during Random control sequences (i.e., when shapes randomly moved).

#### 2.2.6. Definition and operationalization of regions of interest

**Figure 2.**
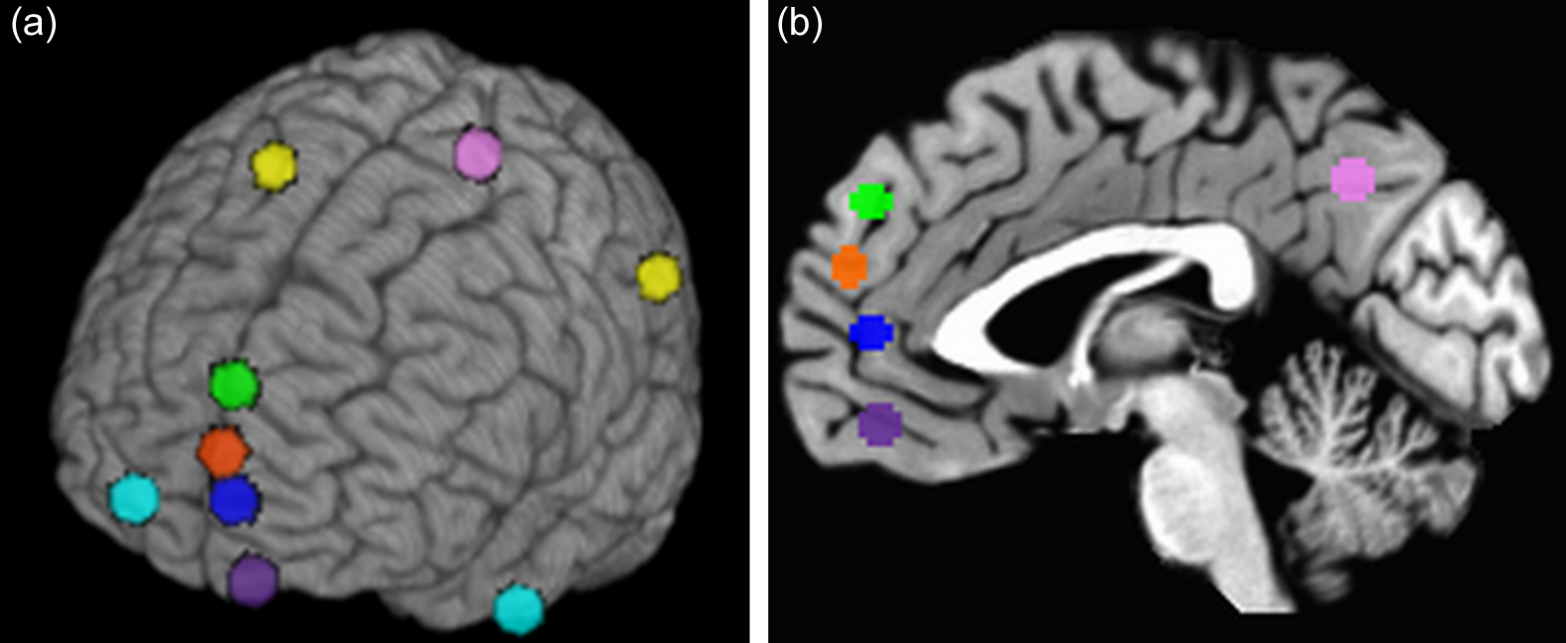
Left and Left-4 Sagittal cut views of a priori regions of interest. Note. Depicted regions include dorsomedial prefrontal cortex (green), medial prefrontal cortex (red), ventromedial prefrontal cortex (blue), orbitofrontal cortex (dark purple); precuneus/posterior cingulate cortex (light purple); right and left temporoparietal junctions (yellow); right and left temporal poles (turquoise).

A priori (regions of interest) ROIs were identified using Van Overwalle’s (2009) meta-analysis of ToM-related brain regions and Neurosynth’s automated lexical meta-analysis software found at https://neurosynth.org/. The search terms “mentalizing” and “theory mind” were used in Neurosynth to identify relevant regions. Selected regions included dorsomedial, medial, and ventromedial prefrontal cortices (dmPFC, mPFC, and vmPFC; separately defined), orbitofrontal cortex (OFC), right and left TPJs (combined), right and left temporal poles (TP; combined), and the precuneus/posterior cingulate cortex (PCC). The SPM12 MarsBar extension was used to generate ROI masks, and the SPM12 lmcalc function was used to combine right and left ROIs into one ROI mask. The sphere radius for each ROI was defined as 5 mm from central coordinates. Coordinates and anatomical labels for each ROI are provided in Table 2 and Figure 2.

**Table 2.** A priori regions of interest

Region of interest	Center of mass coordinates (MNI)		
	*X*	*Y*	*Z*
dmPFC	0	50	35
mPFC	0	55	20
vmPFC	0	50	5
OFC	−4	48	−16
R. and L. TPJ	±50	−55	25
Precuneus/Posterior cingulate (PCC)	0	−60	40
R. & L. TP	±50	8	−30

L, left, MNI, Montreal Neurologic Institute, R, right.

### 2.3. Analyses

#### 2.3.1. Hypothesis testing

Three main sets of analyses were conducted to examine relations between neural activity and connectivity related to ToM and relevant personality and psychopathological constructs. Across analyses, statistical significance was determined using an alpha of *p* < .005 (Benjamin et al., 2018). In addition, for the second and third sets of analyses, a false discovery rate adjustment was planned only in the advent that statistically significant results were found (Benjamini & Hochberg, 1995). For each of the following analyses that used ROIs, the data were averaged across all of the voxels within the ROI and then extracted to generate a single number per ROI per subject. For the *t*-tests, for example, this resulted in a single parameter estimate per subject; for the correlation analyses, this resulted in an average BOLD time series for the ROI.

In the first set of analyses, to confirm the presence of greater activation during ToM sequences versus Random control sequences in a priori ROIs, one-sample *t*-tests were conducted examining whether average group-level contrast activation was different than zero for each whole ROI. In the second set of analyses, fMRI BOLD signal associated with ToM sequences was contrasted with Random sequences to confirm task activation in expected regions. Between-person correlations were then conducted to examine relations between personality domains and psychopathology indices, on the one hand, and BOLD contrasts, on the other, for each a priori ROI, separately.

In the third set of analyses, psychophysiological interaction (PPI) analyses were conducted using SPM12 to investigate the degree to which functional synchrony between regions was dependent on experimental task condition. Specifically, PPI analysis identifies whether two ROIs are more highly correlated in one experimental condition (ToM) versus another (Random). This involves correlating the average time series of a seed ROI with the time series of each voxel of a target ROIs. A PPI interaction coefficient constitutes the degree to which the time series of the seed ROI is differentially correlated with the time series of the target ROI voxels based on the task condition variable. Accordingly, for each participant, six sets of PPI interaction coefficients were generated between each combination of three ROIs (vmPFC, mPFC, OFC, and PCC were excluded due to nonsignificant ToM effects). Across PPI analyses, hemodynamic response was deconvolved onto task conditions in order to control for asynchrony between task design and fMRI hemodynamic response (Gitelman et al., 2003). In addition, to include both fMRI runs (i.e., right to left, left to right), SPM12 specified run as a regressor in PPI analyses. PPI analyses generated an interaction coefficient for each subject indicating the degree to which the synchrony between unique ROI pairs increased (or decreased) in the ToM versus Random condition. ROIs were included in analyses that exhibited significant task-dependent contrast activation in the first set of analyses. Between-person correlations were then conducted between these interaction coefficients and personality/psychopathology variables.

#### 2.3.2. Preregistration note

Analyses were preregistered within the initial abstract submission to *Journal of Personality Neuroscience.* Two deviations from our original preregistered plan are notable. First, our preregistered plan was to examine fine-grained personality aspects in relation to neural processing in order to enhance the prediction of the very specific neural response elicited by the social cognition task. In view of reviewers’ objections to the adequacy of this measurement approach, however, aspect-level analyses were moved to supplemental materials, and domain-level analyses were selected for primary analyses instead. Second, our preregistered plan was to utilize Independent Component analyses to uncover alternative neural regions exhibiting task-dependent activation and then examine these regions in relation to personality variables. This analytic approach, however, was not undertaken due to the authors’ reliance on SPM12.

#### 2.3.3. Power analysis

A power analysis was conducted using G * Power (Faul, Erdfelder, Lang & Buchner, 2007). With *N* = 973, a two-tailed significant value = .005, power estimates ranged from 63% (*r* =.10) to <99.9% (*r* = .20), and a power threshold of 80% was met with an effect size of approximately .12. These estimates suggest adequate power for detecting a small effect size.

## 3. Results

### 3.1. Relations between personality and psychopathology

Pearson correlations were calculated to examine relations between NEO-FFI personality domains and ASR psychopathology indices, while employing a statistical significance threshold of *p* < .005 to control for type I error (Table 3). The pattern of correlations among personality domains was broadly consistent with previous work (e.g., Costa & McCrae, 1992). Internalizing and Externalizing psychopathology indices displayed a large, positive relation as expected (e.g., Kotov et al., 2017). Furthermore, as expected, ASR Internalizing evinced a strong relation with FFM Neuroticism, and ASR Externalizing evinced moderate relations with FFM Antagonism and Disinhibition. Notably, ASR Externalizing was more strongly associated with FFM Antagonism (*r* = .47) than FFM Disinhibition (*r* = .31), suggesting that it may be somewhat more reflective of HiToP Antagonistic Externalizing than Disinhibited Externalizing.^2^

**Table 3.** Correlations between personality domains and psychopathology indices

	1	2	3	4	5	6
1. Neuroticism	–					
2. Extraversion	−.35*	–				
3. Openness	.05	.08	–			
4. Antagonism	.30*	−.28*	−.10*	–		
5. Disinhibition	.42*	−.26*	.14*	.23*	–	
6. Externalizing	.41*	−.01	.13*	.47*	.31*	–
7. Internalizing	.70*	−.38*	.11*	.27*	.32*	.58*

* *p* < .005.

### 3.2. Confirming task-dependent activation in a priori ROIs

To confirm the presence of greater activation during ToM sequences versus Random sequences in a priori ROIs, one-sample *t*-tests were conducted using a statistical significance threshold of *p* < .005. Significant group-level contrast activation was found for dmPFC, right and left TPJ, and right and left TP, but not for vmPFC, mPFC, OFC, or PCC. Mean contrast activation parameter estimates for dmPFC, TPJ, and TP were .24, .27, and .13, respectively.

### 3.3. ROI analyses relating theory of mind-based activation to personality and psychopathology

Pearson correlations were calculated to examine relations between task-dependent activation in a priori ROIs with NEO-FFI personality domains and ASR psychopathology indices. For FFM Antagonism and ASR Externalizing (i.e., two personality/psychopathology variables *X* three ROIs = six analyses total), no significant correlations were observed with task activation in any of the a priori ROIs (range: −.02 < *r* < .01; see Table 4). With respect to exploratory analyses, Pearson correlations were calculated that examined other FFM personality domains and the ASR Internalizing composite, which is found in Table 4 (i.e., five personality/psychopathology variables *X* three ROIs = 15 analyses total). Results indicated no significant correlations with task activation in any a priori ROIs (range: −.04 < *r* < .04). Multivariate analyses in which neural activity in each ROI was regressed on all personality domains simultaneously also resulted in no significant results.

**Table 4.** Correlations between fMRI theory of mind processing, personality, and psychopathology

	Dorsomedial prefrontal	Temporoparietal junction	Temporal pole
Hypothesis-driven variables			
Antagonism	−.02	−.01	−.02
Externalizing	.01	−.01	.00
Exploratory variables			
Internalizing	.01	.00	.00
Neuroticism	.00	−.01	−.02
Extraversion	−.04	.00	.02
Openness	.00	−.03	.04
Disinhibition	.02	−.03	.01

Antagonism = reversed Agreeableness; Disinhibition = reversed Conscientiousness; Internalizing and Externalizing represent psychopathology composites of relevant ASR subscales; *p < .005

### 3.4. Relating differential theory of mind-based synchrony to personality and psychopathology

Pearson correlations were calculated to examine relations between PPI interaction coefficients (i.e., indexing differential connectivity between ROIs based on ToM task condition), on the one hand, and FFM personality domains and ASR psychopathology indices, on the other (see Table 5). For FFM Antagonism and ASR Externalizing (i.e., two personality/psychopathology variables *X* six ROI pairs = 12 analyses total), no statistically significant correlations were observed (range: −.01 < *r* < .07). A second set of correlations were examined containing exploratory variables (i.e., FFM Neuroticism, Extraversion, Openness, Disinhibition, and ASR Internalizing), which is found in Table 5 (i.e., five personality/psychopathology variables *X* six ROI pairs = 30 analyses total). No statistically significant correlations were observed (range: −.06 < *r* < .06). Multivariate analyses in which neural synchrony was regressed on all personality domains resulted in no significant results.

**Table 5.** Correlations between psychophysiological interaction coefficients and NEO-FFI antagonism and externalizing

	Seed: dorsomedial prefrontal		Seed: temporoparietal junction		Seed: temporal pole	
	Temporoparietal junction	Temporal pole	Dorsomedial prefrontal	Temporal pole	Dorsomedial prefrontal	Temporoparietal junction
Hypothesis-driven variables						
Antagonism	.03	.04	.03	.05	.05	.04
Externalizing	.04	.07	−.01	.04	.05	.04
Exploratory variables						
Internalizing	.00	.00	.00	.00	.02	.03
Neuroticism	.02	.04	.03	.01	.02	.06
Extraversion	.03	.02	−.03	−.02	−.04	−.06
Openness	−.03	−.02	−.03	−.02	−.03	.04
Disinhibition	−.05	−.03	−.03	−.02	.00	.05

Antagonism = reversed Agreeableness; Disinhibition = reversed Conscientiousness; Internalizing and Externalizing represent psychopathology composites of relevant subscales; *p < .005

## 4. Discussion

Theoretical links between cognitive ToM, Antagonistic-Externalizing, and FFM Antagonism suggest that psychopathological features related to antisocial behavior, aggression, and rule-breaking tendencies could rest in part on common neurobiologically instantiated social-cognitive substrates. In the present study, our primary aim was to investigate hypothesized relations between externalizing traits/symptoms and neural activity/synchrony during ToM video sequences in a large community sample (*N* = 973). Hypothesized relations were not found across the 72 analyses examining relations between FFM Antagonism and Antagonistic Externalizing and neural activation during the ToM task, across main, exploratory, and supplemental analyses.^3^ These null results suggest that FFM Antagonism and Antagonistic Externalizing psychopathology may not be related to ToM-elicited activity in or synchrony between neural regions linked to ToM. More broadly, our results may show that FFM Antagonism and Antagonistic Externalizing symptoms do not rest on neurobiological substrates related to social-cognitive ToM, and other neural systems are more meaningfully related.

Although the current null results may be reflective of a “true” lack of association between personality traits and neural activation in a ToM task, these null results may also stem from a number of methodological factors. First, reasoning about others’ mental states represents a complex cognitive capacity that has been operationalized in multiple ways and may encompass multiple related but separable constructs that bear varying relevance to personality. Specific operationalizations of ToM have ranged from identifying simple acts of deception (Dziobek et al., 2006) to analyzing complex social attributions and motives (e.g., Pellecchia et al., 2015). The present study used a social cognition task that displayed shapes taking into account each other’s feelings and thoughts. Although these stimuli are likely to elicit cognitive processing of external mental states, it is possible that other facets of social-cognitive ToM exist that differ meaningfully from this operationalized construct and may be more relevant to FFM Antagonism and Antagonistic Externalizing symptoms. Inasmuch as this is the case, the present social cognition task may be limited in eliciting neural ToM processing underlying externalizing forms of psychopathology and related personality traits, and additional functional tasks may be required. For example, it may behoove researchers to investigate differences in neural processing in response to more complex demonstrations of social-cognitive ToM.

On the other hand, if the underlying social-cognitive ToM construct is unified across different operationalizations that have sought to elicit it, it is plausible that our null results emerged because the present social cognition task failed to adequately elicit it. Although our test of task-based contrast activation yielded statistically significant activation in three ROIs, these ROIs represented less than half of the a priori ROIs that we tested and that have been linked empirically to ToM, raising concerns about the task’s validity in eliciting ToM. Even further, it is possible that the social cognition task was valid in distinguishing between neural processing during ToM activity (Mental video sequences) and ToM absence (Random video sequences), but still failed to elicit ToM in a way that would discriminate between participants higher and lower in ToM ability. Our study’s validity depended not only on ToM sequences eliciting greater neural ToM activity on average across people than during Random sequences, but also eliciting greater neural ToM response from individuals higher in ToM ability than those lower in ToM ability. Unfortunately, a measure of ToM ability was not obtained in the present study. The absence of a ToM-related behavioral performance metric was unfortunately quite significant in limiting the interpretation of our results. In consequence, we were unable to determine which of the following conclusions our null results suggested: (a) that the null relation between FFM Antagonism/Antagonistic Externalizing and neural processing during a task designed to elicit ToM reflected a null relation between personality and ToM performance, contrary to prior research; (b) that the null relation reflected that the social cognition task failed to measure ToM adequately, and would therefore not elicit ToM-related neural processing; or (c) that Antagonism/Antagonistic Externalizing and neural activity/synchrony both have relations to ToM, but Antagonism/Antagonistic Externalizing does not show a relation to neural processing during a task designed to elicit ToM, specifically. A fourth (d) possibility that also stems from the properties of the ToM construct is that personality traits, even at more granular structural levels of analysis (e.g., aspects), contain so great a diversity of cognitive, emotional, and behavioral expressions that they do not correlate with very specific neural functions, even if a specific neural function contributes in some small degree to trait patterns. Observing a correlation between the two may be made yet more unlikely when the specific neural function in question is circumscribed by a single, specific cognitive task such as the one used in the present study.

Second, a growing body of research has critically examined the conditions under which fMRI may be validly used to estimate reliable individual differences in neural functioning (e.g., Elliott et al., 2019a; Infantolino, Luking, Sauder, Curtin & Hajcak, 2018). These findings suggest that using contrast estimates and PPI interaction coefficients as valid measures of individual differences in functional activity and connectivity depends on multiple conditions, many of which were not satisfied in the present study, which could contribute to null and/or erroneous results. With respect to between-person estimates of *BOLD contrast estimates*, previous findings indicate that robust effects of within-subject differences in task-based activation of particular ROIs do not necessarily translate into reliable between-subject scores on contrast activation (e.g., Infantolino et al., 2018; Nord, Gray, Charpentier, Robinson & Roiser, 2017). This is important as the latter is considered necessary in order to utilize functional patterns of brain activity as stable biomarkers of dispositional traits. Indeed, Elliott and colleagues (2019b) demonstrated strongly suboptimal test–retest reliability in between-subject contrast activation scores (e.g., intra-class correlation [ICC] range = ~.13 – .37 across ROIs) among HCP participants engaged in the social cognition task paradigm when tested months apart.^4^ Their findings suggest that poor reliability associated with between-subject contrast activation scores is likely to have severely attenuated correlations between Antagonism/Antagonistic Externalizing and ROI activation in the present study. Regrettably, suboptimal psychometric properties of the fMRI social cognition task are consistent with growing field-wide concern surrounding the adequacy of fMRI tasks (Elliott et al., 2019b; Herting, Gautam, Chen, Mezher & Vetter, 2018) and behavioral tasks more broadly (Enkavi et al., 2019).

With respect to the validity of hypothesis-driven analyses examining ToM-related *synchrony* between regions, previous findings suggest that the test–retest reliability of between-subject task-based PPI interaction coefficients is generally low (i.e., ICC < .40 for the majority of whole brain PPI effects for two tested seeds; Di & Biswal, 2017). Elliott and colleagues (2019a) also demonstrated poor test–retest reliability of intrinsic connectivity (though not using PPI analysis) at scan lengths of 5 min or less, which coincides with the present method. While the effect of insufficient test–retest reliability on the validity of the present results is not known, the foregoing findings raise substantial concerns that poor reliability related to the social cognition task paradigm used herein may account for observed null and/or erroneous results. More broadly, these methodological issues may pose significant challenges for the validity of functional individual difference variables that exhibit suboptimal test–retest reliability across contrast and PPI analyses. Indeed, the list of well-powered yet failed efforts to link personality traits to structural and functional neural variables seems to be growing field-wide (e.g., Avinun, Israel, Knodt & Hariri, 2019; Baranger et al., 2020; Gray, Owens, Hyatt & Miller, 2018; Hyatt et al., 2020). Unfortunately, study design attributes that researchers advocate for to support adequate test–retest reliability of functional variables (e.g., >40 min of scan length, large sample size; Elliott et al., 2019a) come with significant financial and temporal costs that may hamper progress in the current technological and economic climate.

A third and final limitation is that the properties of our sample and analytic plan could have contributed to type II error. First, the HCP sample is a relatively healthy community sample that is likely to exhibit relatively low levels of Antagonistic Externalizing psychopathology. The relative range restriction of FFM Antagonism and Antagonistic Externalizing in the present sample could obscure an association between neural ToM processing and Antagonistic Externalizing symptoms in the population. Nevertheless, the dimensional nature of externalizing-related personality disorders and related personality traits suggests that variance in externalizing-related and disagreeable traits within a moderate range of the trait spectrum could still show associations with ToM impairment. Second, ROIs were defined based on the existing empirical literature rather than an empirical examination of regions evincing high neural ToM activity and synchrony in the present sample. It is conceivable that examining Antagonistic Externalizing symptoms in relation to empirically derived ROIs could have yielded effects that were not found in the present examination (although these additional analyses would be associated with elevated risk for Type I error). Third, the validity of supplemental analyses examining narrower personality aspects in relation to neural processing is likely to have been negatively impacted by lower internal consistency on the part of key personality aspects (i.e., Cronbach alpha ranged from .58 to .70 for Antagonism aspects). Examining narrower aspects or facets of personality in future work without sacrificing measurement precision through the use of longer measures of personality/psychopathology is encouraged.

## 5. Conclusion

The present study leverages the largest sample to date and an fMRI social cognition task paradigm to examine the degree to which neural ToM functioning underlies FFM Antagonism and Antagonistic Externalizing psychopathology. Although a study with these methodological strengths confers some confidence in interpreting null results, results should nevertheless be interpreted with caution in view of important methodological considerations, namely the relevance of the social cognition task operationalization of ToM to higher-order behavioral and personality features and low test–retest reliability associated with the social cognition task. The present study may provide evidence that neural ToM functioning does not underlie Antagonistic Externalizing symptoms or associated personality traits. Even so, the absence of positive results and miniscule effect sizes could be reflective of concerns related to the viability of the social cognition task, fMRI-based biomarkers of ToM, and fMRI task paradigms with similarly suboptimal reliability. These concerns make further efforts at replication critical and may raise the importance of testing different operationalizations of ToM and designing ToM tasks that sustain ToM processing for longer periods of time, in accordance with evidence that longer scan lengths confer stronger test–retest reliability within the context of investigations of functional synchrony/connectivity (Elliott et al., 2019a).

## Footnotes

1 For comprehensiveness, we also tested narrower personality aspects (based on the Big Five aspects model [BFAS; DeYoung, Quilty & Peterson, 2007]) and Exploratory Factor Analysis-derived factors comprised of items from measures of personality and psychopathology in relation to neural activity and synchrony during the ToM paradigm (presented in online supplemental material).

2 This difference in effect size was confirmed using Steiger’s (1980) test (*z* = 4.55, *p* < .005).

3 One significant correlation was found between neural synchrony involving the dPFC and TP and an Antagonistic Externalizing factor extracted from exploratory factor analysis of all NEO-FFI and ASR items (*r* = .09; see online supplemental materials; Table S9). We however do not interpret this result as it is likely to have emanated from Type I error associated with a statistical significance threshold of .005, which permits Type I error in approximately .5% of analyses, equating to 1.22 of cases among the 243 main, exploratory, and supplemental analyses devoted to examining relations with neural activity and synchrony. This result was also no longer significant at *p* < .005 after adjusting for the false discovery rate (Benjamini & Hochberg, 1995).

4 Though still suboptimal, it bears noting that the anterior temporal lobe (similar to the temporal lobe examined in the present study) exhibited reliability exceeding .30. Reliability for an ROI approximating the posterior cingulate cortex was not examined.

## Supplementary material

For supplementary material accompanying this paper visit https://doi.org/10.1017/pen.2020.12. click here to view supplementary material

## References

[ref1] Achenbach, T. M. , & Rescorla, L. A. (2003). Manual for the ASEBA adult forms & profiles. Burlington: VT University of Vermont, Research Centre for Children, Youth & Families.

[ref2] Allen, T. A. , Rueter, A. R. , Abram, S. V. , Brown, J. S. , & DeYoung, C. G. (2017). Personality and neural correlates of mentalizing ability. European Journal of Personality, 31, 599–613. 10.1002/per.2133 29610548 PMC5877481

[ref3] Avinun, R. , Israel, S. , Knodt, A. R. , & Hariri, A. R. (2019). Little evidence for associations between the Big Five personality traits and variability in brain gray or white matter. bioRxiv, 1–31. 10.1101/658567 PMC7593529 32599267

[ref4] Barch, D. M. , Burgess, G. C. , Harms, M. P. , Petersen, S. E. , Schlaggar, B. L. , Corbetta, M. , … Van Essen, D. C. (2013). Function in the human connectome: Task-fMRI and individual differences in behavior. NeuroImage, 80, 169–189. 10.1016/j.neuroimage.2013.05.033 23684877 PMC4011498

[ref5] Baranger, D. A. , Few, L. R. , Sheinbein, D. H. , Agrawal, A. , Oltmanns, T. F. , Knodt, A. R. , … Bogdan, R. (2020). Borderline personality traits are not correlated with brain structure in two large samples. Biological Psychiatry: Cognitive Neuroscience and Neuroimaging, 5, 669–677. 10.1016/j.bpsc.2020.02.006 32312691 PMC7360105

[ref6] Benjamini, Y. , & Hochberg, Y. (1995). Controlling the false discovery rate: A practical and powerful approach to multiple testing. Journal of the Royal Statistical Society: Series B (Methodological), 57, 289–300. 10.1111/j.2517-6161.1995.tb02031.x

[ref7] Benjamin, D. J. , Berger, J. O. , Johannesson, M. , Nosek, B. A. , Wagenmakers, E.-J. , Berk, R. , … Johnson, V. E. (2018). Redefine statistical significance. Nature Human Behavior, 2, 6–10. 10.1038/s41562-017-0189-z 30980045

[ref8] Blair, R. J. R. , Sellars, C. , Strickland, I. , Clark, F. , Williams, A. , Smith, M. , & Jones, L. (1996). Theory of mind in the psychopath. Journal of Forensic Psychiatry, 7, 15–25. 10.1080/09585189608409914

[ref9] Castelli, F. , Frith, C. , Happe, F. , & Frith, U. (2002). Autism, Asperger syndrome and brain mechanisms for the attribution of mental states to animated shapes. Brain, 125, 1839–1849. 10.1093/brain/awf189 12135974

[ref10] Castelli, F. , Happe, F. , Frith, U. , & Frith, C. (2000). Movement and mind: A functional imaging study of perception and interpretation of complex intentional movement patterns. Neuroimage, 12, 314–325. 10.1006/nimg.2000.0612 10944414

[ref11] Costa, P. T. , & McCrae, R. R. (1992). Revised NEO Personality Inventory (NEO PI-R) and NEO Five-Factor Inventory (NEO-FFI) Professional Manual. Lutz, FL: Psychological Assessment Resources, Inc.

[ref12] Cuthbert, B. N. , & Insel, T. R. (2013). Toward the future of psychiatric diagnosis: The seven pillars of RDoC. BMC Medicine, 11, 1–8. 10.1186/1741-7015-11-126 23672542 PMC3653747

[ref13] DeYoung, C. G. , & Gray, J. R. (2009). Personality neuroscience: Explaining individual differences in affect, behavior, and cognition. In P. J. Corr & G. Matthews (Eds.), The Cambridge handbook of personality (pp. 323–346), New York: Cambridge University Press.

[ref14] DeYoung, C. G. , Quilty, L. C. , & Peterson, J. B. (2007). Between facets and domains: Ten aspects of the Big Five. Journal of Personality and Social Psychology, 93, 880–896. 10.1037/0022-3514.93.5.880 17983306

[ref15] Di, X. , & Biswal, B. B. (2017). Psychophysiological interactions in a visual checkerboard task: Reproducibility, reliability, and the effects of deconvolution. Frontiers in Neuroscience, 1–16. 10.3389/fnins.2017.00573 29089865 PMC5651039

[ref16] Drayton, L. A. , Santos, L. R. , & Baskin-Sommers, A. (2018). Psychopaths fail to automatically take the perspective of others. Proceedings of the National Academy of Sciences of the United States of America, 115, 3302–3307. 10.1073/pnas.1721903115 29531085 PMC5879707

[ref17] Dziobek, I. , Fleck, S. , Kalbe, E. , Rogers, K. , Hassenstab, J. , Brand, M. , … Convit, A. (2006). Introducing MASC: A movie for the assessment of social cognition. Journal of Autism and Developmental Disorders, 36, 623–636. 10.1007/s10803-006-0107-0 16755332

[ref18] Elliott, M. L. , Knodt, A. R. , Cooke, M. , Kim, M. J. , Melzer, T. R. , Keenan, R. , … Hariri, A. R. (2019a). General functional connectivity: Shared features of resting-state and task fMRI drive reliable and heritable individual differences in functional brain networks. NeuroImage, 189, 516–532. 10.1016/j.neuroimage.2019.01.068 30708106 PMC6462481

[ref19] Elliott, M. L. , Knodt, A. R. , Ireland, D. , Morris, M. L. , Ramrakha, S. , Sison, M. L. , … Hariri, A. R. (2019b). Poor test-retest reliability of task-fMRI: New empirical evidence and a meta-analysis. BioRxiv, 919, 1–31. 10.1101/681700 PMC7370246 32489141

[ref20] Enkavi, A. Z. , Eisenberg, I. W. , Bissett, P. G. , Mazza, G. L. , MacKinnon, D. P. , Marsch, L. A. , & Poldrack, R. A. (2019). Large-scale analysis of test–retest reliabilities of self-regulation measures. Proceedings of the National Academy of Sciences, 116, 5472–5477. 10.1073/pnas.1818430116 PMC6431228 30842284

[ref21] Ewing, D. , Zeigler-Hill, V. , & Vonk, J. (2016). Spitefulness and deficits in the social-perceptual and social-cognitive components of Theory of Mind. Personality and Individual Differences, 91, 7–13. 10.1016/j.paid.2015.11.050

[ref22] Faul, F. , Erdfelder, E. , Lang, A.-G. , & Buchner, A. (2007). G*Power 3: A flexible statistical power analysis program for the social, behavioral, and biomedical sciences. Behavior Research Methods, 39, 175–191. 10.3758/bf03193146 17695343

[ref23] Fonagy, P. , Target, M. , Steele, H. , & Steele, M. (1998). Reflective functioning manual, version 5.0, for application to Adult Attachment Interviews. London, UK: University College London.

[ref24] Fossati, A. , Somma, A. , Krueger, R. F. , Markon, K. E. , & Borroni, S. (2017). On the relationships between DSM-5 dysfunctional personality traits and social cognition deficits: A study in a sample of consecutively admitted Italian psychotherapy patients. Clinical Psychology & Psychotherapy, 24, 1421–1434. 10.1002/cpp.2091 28493518

[ref25] Gitelman, D. R. , Penny, W. D. , Ashburner, J. , & Friston, K. J. (2003). Modeling regional and psychophysiologic interactions in fMRI: The importance of hemodynamic deconvolution. NeuroImage, 19, 200–207. 10.1016/s1053-8119(03)00058-2 12781739

[ref26] Glasser, M. F. , Sotiropoulos, S. N. , Wilson, J. A. , Coalson, T. S. , Fischl, B. , Andersson, J. L. , … Jenkinson, M. , for the WU-Minn HCP Consortium. (2013). The minimal preprocessing pipelines for the Human Connectome Projects. NeuroImage, 80, 105–124. 10.1016/j.neuroimage.2013.04.127 23668970 PMC3720813

[ref27] Gray, J. C. , Owens, M. M. , Hyatt, C. S. , & Miller, J. D. (2018). No evidence for morphometric associations of the amygdala and hippocampus with the five-factor model personality traits in relatively healthy young adults. PLoS ONE, 13, 10.1371/journal.pone.0204011 PMC6147458 30235257

[ref28] Haas, B. W. , Anderson, I. W. , & Filkowski, M. M. (2015a). Interpersonal reactivity and the attribution of emotional reactions. Emotion, 15, 390–398. 10.1037/emo0000053 25706827

[ref29] Haas, B. W. , Ishak, A. , Denison, L. , Anderson, I. , & Filkowski, M. M. (2015b). Agreeableness and brain activity during emotion attribution decisions. Journal of Research in Personality, 57, 26–31. 10.1016/j.jrp.2015.03.001

[ref30] Herting, M. M. , Gautam, P. , Chen, Z. , Mezher, A. , & Vetter, N. C. (2018). Test-retest reliability of longitudinal task-based fMRI: Implications for developmental studies. Developmental Cognitive Neuroscience, 33, 17–26. 10.1016/j.dcn.2017.07.001 29158072 PMC5767156

[ref31] Hyatt, C. S. , Hallowell, E. S. , Owens, M. M. , Weiss, B. M. , Sweet, L. H. , & Miller, J. D. (2020). An fMRI investigation of the relations between Extraversion, internalizing psychopathology, and neural activation following reward receipt in the Human Connectome Project sample. Personality Neuroscience, 3, e13. 10.1017/pen.2020.11 33354651 PMC7737192

[ref32] Infantolino, Z. P. , Luking, K. R. , Sauder, C. L. , Curtin, J. J. , & Hajcak, G. (2018). Robust is not necessarily reliable: From within-subjects fMRI contrasts to between-subjects comparisons. NeuroImage, 173, 146–152. 10.1016/j.neuroimage.2018.02.024 29458188 PMC5912348

[ref33] Kotov, R. , Krueger, R. F. , Watson, D. , Achenbach, T. M. , Althoff, R. R. , Bagby, R. M. , … Zimmerman, M. (2017). The Hierarchical Taxonomy of Psychopathology (HiTOP): A dimensional alternative to traditional nosologies. Journal of Abnormal Psychology, 126, 454–477. 10.1037/abn0000258 28333488

[ref34] Meinhardt-Injac, B. , Daum, M. M. , Meinhardt, G. , & Persike, M. (2018). The two-systems account of theory of mind: Testing the links to social-perceptual and cognitive abilities. Frontiers in Human Neuroscience, 12, Article ID 25. 10.3389/fnhum.2018.00025 PMC5797799 29445336

[ref35] Lynam, D. R. , Gaughan, E. T. , Miller, J. D. , Mullins-Sweatt, S. , & Widiger, T. A. (2011). Assessing the basic traits associated with psychopathy: Development and validation of the Elemental Psychopathy Assessment. Psychological Assessment, 23, 108–124. 10.1037/a0021146 21171784

[ref36] Nentjes, L. , Bernstein, D. P. , Arntz, A. , Slaats, M. E. , & Hannemann, T. (2015). Theory of mind, social desirability, and unlikely symptom reporting in offenders with and without psychopathy. The Journal of Nervous and Mental Disease, 203, 596–603. 10.1097/NMD.0000000000000335 26164424

[ref37] Nettle, D. , & Liddle, B. (2008). Agreeableness is related to social-cognitive, but not social-perceptual, theory of mind. European Journal of Personality, 22, 323–335. 10.1002/per.672

[ref38] Nord, C. L. , Gray, A. , Charpentier, C. J. , Robinson, O. J. , & Roiser, J. P. (2017). Unreliability of putative fMRI biomarkers during emotional face processing. NeuroImage, 156, 119–127. 10.1016/j.neuroimage.2017.05.024 28506872 PMC5553850

[ref39] O’Connor, B. P. (2005). A search for consensus on the dimensional structure of personality disorders. Journal of Clinical Psychology, 61, 323–345. 10.1002/jclp.20017 15468325

[ref40] Pellecchia, G. , Moroni, F. , Carcione, A. , Colle, L. , Dimaggio, G. , Nicolò, G. , … Semerari, A. (2015). Metacognition assessment interview: Instrument description and factor structure. Clinical Neuropsychiatry, 12, 157–165.

[ref41] Sabbagh, M. A. (2004). Understanding orbitofrontal contributions to theory-of-mind reasoning: Implications for autism. Brain and Cognition, 55, 209–219. 10.1016/j.bandc.2003.04.002 15134854

[ref42] Saulsman, L. M. , & Page, A. C. (2004). The five-factor model and personality disorder empirical literature: A meta-analytic review. Clinical Psychology Review, 23, 1055–1085. 10.1016/j.cpr.2002.09.001 14729423

[ref43] Saxe, R. , & Kanwisher, N. (2003). People thinking about thinking people - The role of the temporo-parietal junction in “theory of mind.” Neuroimage, 19, 1835–1842. 10.1016/S1053-8119(03)00230-1 12948738

[ref44] Spreng, R. N. , & Andrews-Hanna, J. R. (2015). The default network and social cognition. Brain Mapping: An Encyclopedic Reference, 1316, 165–169. 10.1038/s41562-017-0244-9

[ref45] Spunt, R. P. , & Lieberman, M. D. (2012). Dissociating modality-specific and supramodal neural systems for action understanding. Journal of Neuroscience, 32, 3575–3583. 10.1523/JNEUROSCI.5715-11.2012 22399779 PMC6621054

[ref46] Steiger, J. H. (1980). Tests for comparing elements of a correlation matrix. Psychological Bulletin, 87, 245–251. 10.1037/0033-2909.87.2.245

[ref47] Van Essen, D. C. , Smith, S. M. , Barch, D. M. , Behrens, T. E. , Yacoub, E. , Ugurbil, K. , & Wu-Minn HCP Consortium. (2013a). The WU-Minn human connectome project: An overview. Neuroimage, 80, 62–79. 10.1016/j.neuroimage.2013.05.041 23684880 PMC3724347

[ref48] Van Essen, D. C. , Ugurbil, K. , Auerbach, E. , Barch, D. , Behrens, R. , Chang, A. , … Yacoub, E. WU-Minn HCP Consortium (2013b). The human connectome project: A data acquisition perspective. Neuroimage, 62, 2222–2231. 10.1016/j.neuroimage.2012.02.018 PMC3606888 22366334

[ref49] Van Overwalle, F. (2009). Social cognition and the brain: A meta-analysis. Human Brain Mapping, 30, 829–858. 10.1002/hbm.20547 18381770 PMC6870808

[ref50] Vonk, J. , Zeigler-Hill, V. , Ewing, D. , Mercer, S. , & Noser, A. E. (2015). Mindreading in the dark: Dark personality features and theory of mind. Personality and Individual Differences, 87, 50–54. 10.1016/j.paid.2015.07.025

[ref51] Wheatley, T. , Milleville, S. C. , & Martin, A. (2007). Understanding animate agents: Distinct roles for the social network and mirror system. Psychological Science, 18, 469–474. 10.1111/j.1467-9280.2007.01923.x 17576256

[ref52] White, J. S. , Coniston, D. , Rogers, R. , & Frith, U. (2011). Developing the Frith–Happé animations: A quick and objective test of Theory of Mind for adults with autism. Autism Research, 4, 149–154. 10.1002/aur.174 21480540

[ref53] Widiger, T. A. , Lynam, D. R. , Miller, J. D. , & Oltmanns, T. F. (2012). Measures to assess maladaptive variants of the five-factor model. Journal of Personality Assessment, 94, 450–455. 10.1080/00223891.2012.677887 22519804

[ref54] Young, L. , Dodell-Feder, D. , & Saxe, R. (2010). What gets the attention of the temporo-parietal junction? An fMRI investigation of attention and theory of mind. Neuropsychologia, 48, 2658–2664. 10.1016/j.neuropsychologia.2010.05.012 20470808

