# Supplementary material for: Investigating the neural substrates of Antagonistic Externalizing and social-cognitive Theory of Mind: an fMRI examination of functional activity and synchrony
Source: Personal Neurosci. 2021 Jan 19;4:e1. doi: 10.1017/pen.2020.12 (PMC8057509; doi:10.1017/pen.2020.12)
Supplement: Supplementary file 1 [file S2513988620000127sup001.docx]

**Supplementary Materials**

**Examining Relations between Personality Aspects and Neural Processing**

**Derivation of aspect structure**. In order to examine relations between personality and neural functioning with higher resolution, NEO-FFI domains were divided into more granular aspects according to DeYoung, Quilty, and Peterson’s (2007) Big Five Aspects Scale taxonomy. The BFAS aspect-level of structural differentiation has provided good model fit to International Personality Item Pool (IPIP; Goldberg, 1999) data in previous work (DeYoung, Quilty, & Peterson, 2007), and may be well-suited to a neurobiological context as aspects (versus FFM domains) (a) show evidence of better accounting for genetic factors observed to underlie personality (e.g., Jang et al., 2006), and (b) intra-domain aspects have shown differential relations with neurobiological patterns of activation and cognitive domains (e.g., DeYoung & Gray, 2009). Meta-analytic work from Judge and colleagues (2013) has furthermore demonstrated that FFM facets tend to load onto BFAS aspects in reliable ways that closely match DeYoung, Quilty, and Peterson’s (2007) original observations of structural organization. Notably, extant NEO-FFI data was included in this meta-analysis, making it directly relevant to the present study. To establish that the 10-factor aspect model was a good fit, we estimated five hierarchical confirmatory factor analytic (CFA) models in which NEO-FFI items relating to each FFM domain loaded onto FFM facets, and FFM facets loaded onto two related aspects. Correspondence between FFM facets and BFAS aspects was based on meta-analytic work by Judge and colleagues (2013). Models for Neuroticism, Extraversion, Openness, and Disinhibition (i.e., Conscientiousness) showed adequate model fit (see Table S1 for fit indices). Table S2 presents the CFA models we specified and the resulting loadings.

A few deviations from previous BFAS models are notable, however. First, our results did not reflect DeYoung and colleagues’ (2007) observation of a meaningful loading by FFM facet Excitement-seeking on BFAS Enthusiasm. As such, this facet was not specified as an indicator of BFAS Enthusiasm in the factor analytic model for Extraversion or as a contributor to participant scores on BFAS Enthusiasm. Second, the model for Antagonism (i.e., Agreeableness) yielded a negative eigenvalue for the BFAS (Im)Politeness latent factor. As such, exploratory factor analyses (EFA; using principal axis factoring and promax rotation) were conducted to determine an adequately fitting model. Preliminary factor analyses indicated that NEO-FFI item 19 (i.e., involving cooperativeness versus competition) exhibited factor loadings below .30 across multiple models, prompting its removal from subsequent analyses. Parallel analysis on the remaining 11 items indicated the presence of five factors (Horn, 1965); however, closer examination of the parallel analysis plot indicated that the latter two factors did not explain meaningful variance beyond simulated factors. As such, models containing up to three factors were considered. Velicer’s Minimum average partial (MAP; Velicer, 1976) achieved a minimum of .02 with one factor. However, although RMSEA and SRMR values indicated good fit for nearly all models, the close-fit standard recommended by Hu and Bentler (1999) was not reached until three factors were identified (i.e., RMSEA < .06; SRMR < .08). An examination of RMSEA 95% confidence intervals indicated significant improvement in RMSEA in moving from two to three factors (i.e., confidence intervals were not overlapping). A three-factor structure was further supported by (a) the parallel analysis plot, which demonstrated minimal additional variance explained by the four-, five-factor models beyond simulated structures; and (b) the Bayesian Information Criterion (BIC), which achieved a minimum with three factors (Raftery, 1995).

These results provided support for an alternative three-factor structure of NEO-FFI Antagonism (Table S3 for item loadings). The first factor captures selfishness, calculation, and combativeness (subsequently referred to as Selfish); the second factor captures distrusting guardedness (subsequently referred to as Guarded); and the third factor captures inconsiderateness (subsequently referred to as Inconsiderate), and may represent a method factor, as it contains items in the direction of Agreeableness. Factors were moderately to strongly associated, with correlations of .38 (Guarded/Inconsiderate), .60 (Selfish/Inconsiderate), and .64 (Selfish/Guarded). The factor scores, generated from the EFA using Thurstone regression-based weighting, were saved and examined within analyses.

***Internal-consistency.*** Internal-consistency was assessed using Cronbach alpha, ranging from .60 (Assertiveness) to .84 (Withdrawal). Internal-consistency for EFA-derived NEO-FFI Antagonism factors were calculated based on items loading most strongly on each factor. Results ranged from .58 (Guarded) to .70 (Selfish). One of the Big 10 Aspects for Neuroticism was captured using just one item given the limited number of items made available by the NEO-FFI. To assess reliability for this aspect, we found an estimate of the corresponding item’s (*i.e.,* N8 “I often get angry at the way people treat me.”) factor loading onto the Neuroticism factor (Egan, Deary, & Austin, 2000), and squared this loading resulting in a reliability of .34.

**Results.** Table S4 presents correlations among personality aspect predictors. Table S5 and S6 present correlations between personality aspects and neural activity/synchrony. No correlations were statistically significant (*p* < .005).

In addition, in an attempt to partially replicate Allen and colleagues’ (2017) exploratory examination of honesty in relation to neural synchrony, we examined the degree to which a single NEO-FFI item indexing willingness to manipulate was associated with relevant neural activity and synchrony (Tables S5-S6). No statistically significant effects emerged.

**Examining Relations between Combined Personality/Psychopathology Factors and Neural Processing**

**Derivation of combined personality/psychopathology factors.** In view of structural overlap between models of FFM personality and psychopathology (e.g., Kotov et al., 2017), NEO-FFI and Achenbach scale items were factor-analyzed together to produce synthetic factors. EFA analyses (using principal axis factoring and promax rotation) were conducted to determine the best fitting model. Parallel analysis indicated the presence of 17 factors (Horn, 1965); however, closer examination of the parallel analysis plot indicated that only 10 factors explained meaningful variance beyond simulated factors. As such, models containing up to ten factors were considered. Velicer’s Minimum average partial (MAP; Velicer, 1976) achieved a minimum of .002 with ten factors. All models met the close-fit standard recommended by Hu and Bentler (1999) (i.e., RMSEA < .06; SRMR < .08). Scree plot observations suggested a plateau in eigenvalue following five factors, and the five-factor solution furthermore yielded coherent and interpretable factors that accorded well with the HiTop spectra. Six- and seven-factor solutions were also considered but were disfavored due to the presence of narrow incremental factors that yielded minimal incremental variance explained and low item-factor loadings (e.g., λ < .40).

Table S7 presents item loadings onto the five factors. The first factor captures general Neuroticism comprised of Achenbach Anxious/Depressed and Somatic subscale items, a few Achenbach Aggression subscale items indexing mood lability, and NEO-FFI Neuroticism items (subsequently referred to as Negative Affectivity). The second factor captures Achenbach Externalizing items and NEO-FFI Agreeableness items (subsequently referred to as Antagonistic Externalizing). The third factor captures some Achenbach Intrusive and Withdrawn subscale items, and NEO-FFI Extraversion items (subsequently referred to as Extraversion). The fourth factor captures NEO-FFI Conscientiousness items (subsequently reverse-scored and referred to as Disinhibition). The fifth factor captures NEO-FFI Openness items (subsequently referred to as Openness). These results notably reflect the FFM structure of personality, and accord with the HiTOP spectra. The results also reveal substantive heterogeneity within the Achenbach Aggression subscale, with some Aggression items loading more strongly onto the Negative Affectivity factor, and some Intrusive items loading more strongly onto the Extraversion factor, and suggest that Achenbach Externalizing largely reflects Antagonistic Externalizing rather than Disinhibited Externalizing. Negative Affectivity and Antagonistic Externalizing were moderately correlated (*r* = .42), whereas all other inter-factor correlations were small (|.02| < *r* < |.27|). The factor scores, generated from the EFA using Thurstone regression-based weighting, were saved and examined within analyses.

***Internal-consistency.*** Internal-consistency for EFA-derived factors were calculated based on items loading most strongly on each factor. Results ranged from .77 (Openness) to .91 (Negative Affectivity).

**Results.** Table S7 presents correlations among factors. Table S8 and S9 present correlations between factors and neural activity/synchrony. Only one correlation was statistically significant, namely between the Antagonistic Externalizing factor and neural synchrony between the dPFC and TP (*r* = .09, *p* < .005). Given this study’s Type I error rate, this result was not deemed interpretable. See Discussion for full interpretation of this result.

**References**

DeYoung, C. G., Quilty, L. C. and Peterson, J. B. (2007). Between facets and domains: Ten aspects of the Big Five, *Journal of Personality and Social Psychology, 93*, 880–896. Retrieved from http://search.ebscohost.com.proxy-remote.galib.uga.edu/login.aspx?direct=true&db=edsbl&AN=RN217995898&site=eds-live

Egan, V., Deary, I., & Austin, E. (2000). The NEO-FFI: Emerging British norms and an item-level analysis suggest N, A and C are more reliable than O and E. *Personality and Individual Differences, 29*, 907-920. Retrieved from http://search.ebscohost.com.proxy-remote.galib.uga.edu/login.aspx?direct=true&db=edsbl&AN=RN080738250&site=eds-live

Goldberg, L. R. (1999). A broad-bandwidth, public domain, personality inventory measuring the lower-level facets of several five-factor models. In I. Mervielde, I. Deary, F. De Fruyt, & F. Ostendorf (Eds.), *Personality psychology in Europe* (Vol. 7, pp. 7–28). Tilburg, the Netherlands: Tilburg University Press.

Horn, J.L. (1965) A rationale and test for the number of factors in factor analysis. *Psychometrika, 30*, 179-185. Retrieved from http://search.ebscohost.com.proxy-remote.galib.uga.edu/login.aspx?direct=true&db=edb&AN=71555376&site=eds-live

Hu, L.T., & Bentler, P. M. (1999). Cutoff criteria for fit indexes in covariance structure analysis: Conventional criteria versus new alternatives. *Structural Equation Modeling, 6*, 1-55. Retrieved from http://search.ebscohost.com.proxy-remote.galib.uga.edu/login.aspx?direct=true&db=edsbl&AN=RN054352694&site=eds-live

Jang, K. L., Livesley, W. J., Ando, J., Yamagata, S., Suzuki, A., Angleitner, A., … Spinath, F. (2006). Behavioral genetics of the higher-order factors of the Big Five. *Personality and Individual Differences, 41*, 261–272. https://doi-org.proxy-remote.galib.uga.edu/10.1016/j.paid.2005.11.033

Judge, T. A., Rodell, J. B., Klinger, R. L., Simon, L. S., & Crawford, E. R. (2013). Hierarchical representations of the five-factor model of personality in predicting job performance: Integrating three organizing frameworks with two theoretical perspectives. *Journal of Applied Psychology, 98*, 875–925. https://doi-org.proxy-remote.galib.uga.edu/10.1037/a0033901

Raftery, A. E. (1995). Bayesian model selection in social research. *Sociological Methodology, 25,* 111-163. https://doi-org.proxy-remote.galib.uga.edu/10.2307/271063

Velicer, W. F. (1976) Determining the number of components from the matrix of partial correlations. *Psychometrika,* *41*, 321–327. Retrieved from http://search.ebscohost.com.proxy-remote.galib.uga.edu/login.aspx?direct=true&db=edb&AN=71556225&site=eds-live

**Supplemental Tables**

Table S1

| Model fit results for BFAS aspect models | | |  |  |  |  |
| --- | --- | --- | --- | --- | --- | --- |
| Model | *X^2^* | df | CFI | TLI | RMSEA | SRMR |
| Neuroticism | 253.72* | 52 | .94 | .92 | .063 | .04 |
| Extraversion | 268.67* | 49 | .91 | .87 | .068 | .05 |
| Openness | 221.90* | 49 | .93 | .90 | .060 | .05 |
| Conscientiousness | 188.65* | 45 | .95 | .93 | .057 | .04 |
| Note. df = degrees of freedom; **p* < .05 | | |  |  |  |  |

Table S2

| Confirmatory Factor loadings for BFAS aspect models | | | | | |
| --- | --- | --- | --- | --- | --- |
| Item Number | Specified Facet | λ | Facet | Specified Aspect | λ |
| Lower-order structure | | | Higher-order structure | | |
| Confirmatory Model for Neuroticism | | | | | |
| NEO FFI 21 | N1 Anxiety | .61 | N1 Anxiety | Withdrawal | .84 |
| NEO FFI 01 (R) | N1 Anxiety | .58 | N3 Depression | Withdrawal | 1.00 |
| NEO FFI 31 (R) | N1 Anxiety | .75 | N6 Vulnerability | Withdrawal | .98 |
| NEO FFI 36 | N2 Angry Hostility | .92 | N4 Self-consciousness | Withdrawal | 1.00 |
| NEO FFI 26 | N3 Depression | .74 | N6 Vulnerability | Withdrawal | 1.00 |
| NEO FFI 41 | N3 Depression | .60 |  |  |  |
| NEO FFI 16 (R) | N3 Depression | .61 |  |  |  |
| NEO FFI 46 (R) | N3 Depression | .54 |  |  |  |
| NEO FFI 06 | N4 Self-consciousness | .50 |  |  |  |
| NEO FFI 56 | N4 Self-consciousness | .59 |  |  |  |
| NEO FFI 11 | N6 Vulnerability | .73 |  |  |  |
| NEO FFI 51 | N6 Vulnerability | .46 |  |  |  |
| Confirmatory Model for Extraversion | | | | | |
| NEO FFI 17 | E1 Warmth | .79 | E1 Warmth | Enthusiasm | .68 |
| NEO FFI 02 | E2 Gregariousness | .72 | E2 Gregariousness | Enthusiasm | .82 |
| NEO FFI 27 (R) | E2 Gregariousness | .54 | E6 Positive Emotions | Enthusiasm | .72 |
| NEO FFI 57 (R) | E3 Assertiveness | 1.00 | E3 Assertiveness | Assertiveness | .44 |
| NEO FFI 32 | E4 Activity | .46 | E4 Activity | Assertiveness | .83 |
| NEO FFI 47 | E4 Activity | .47 | E5 Excitement-seeking | Assertiveness | .53 |
| NEO FFI 52 | E4 Activity | .53 |  |  |  |
| NEO FFI 22 | E5 Excitement-seeking | .88 |  |  |  |
| NEO FFI 07 | E6 Positive Emotions | .39 |  |  |  |
| NEO FFI 37 | E6 Positive Emotions | .66 |  |  |  |
| NEO FFI 12 (R) | E6 Positive Emotions | .39 |  |  |  |
| NEO FFI 42 (R) | E6 Positive Emotions | .67 |  |  |  |
| Confirmatory Model for Openness | | | | | |
| NEO FFI 03 (R) | O1 Fantasy | 1.00 | O1 Fantasy | Aesthetic Openness | .31 |
| NEO FFI 13 | O2 Aesthetics | .77 | O2 Aesthetics | Aesthetic Openness | .79 |
| NEO FFI 43 | O2 Aesthetics | .72 | O3 Feelings | Aesthetic Openness | .32 |
| (R)NEO FFI 23 | O2 Aesthetics | .61 | O4 Actions | Aesthetic Openness | 1.00 |
| (R)NEO FFI 33 | O3 Feelings | .90 | O6 Values | Aesthetic Openness | .57 |
| NEO FFI 28 | O4 Actions | .35 | O5 Ideas | Intellect | 1.00 |
| NEO FFI 08 (R) | O4 Actions | .13 |  |  |  |
| NEO FFI 53 | O5 Ideas | .42 |  |  |  |
| NEO FFI 58 | O5 Ideas | .71 |  |  |  |
| NEO FFI 48 (R) | O5 Ideas | .69 |  |  |  |
| NEO FFI 18 (R) | O6 Values | .42 |  |  |  |
| NEO FFI 38 (R) | O6 Values | .66 |  |  |  |
| Confirmatory Model for Conscientiousness | | | | | |
| NEO FFI 05 | C2 Order | .56 | C2 Order | Orderliness | .66 |
| NEO FFI 15 (R) | C2 Order | .26 | C3 Dutifulness | Orderliness | .85 |
| NEO FFI 55 (R) | C2 Order | .76 | C4 Achievement Striving | Industriousness | .83 |
| NEO FFI 20 | C3 Dutifulness | .25 | C5 Self-discipline | Industriousness | 1.00 |
| NEO FFI 40 | C3 Dutifulness | .48 |  |  |  |
| NEO FFI 45 (R) | C3 Dutifulness | .81 |  |  |  |
| NEO FFI 25 | C4 Achievement Striving | .55 |  |  |  |
| NEO FFI 35 | C4 Achievement Striving | .47 |  |  |  |
| NEO FFI 60 | C4 Achievement Striving | .42 |  |  |  |
| NEO FFI 10 | C5 Self-discipline | .55 |  |  |  |
| NEO FFI 50 | C5 Self-discipline | .45 |  |  |  |
| NEO FFI 30 (R) | C5 Self-discipline | .53 |  |  |  |
| Note. N = Neuroticism; E = Extraversion; O = Openness; C = Conscientiousness; R = reverse-coded;  λ = loading value. | | | | | |

Table S3

| Exploratory factor analysis of NEO-FFI Agreeableness Item loadings | | | |
| --- | --- | --- | --- |
| Items | Selfish | Guarded | Inconsiderate |
| NEO-FFI 24 (R) | .24 | **.42** | - |
| NEO-FFI 29 (R) | -.18 | **.86** | - |
| NEO-FFI 39 (R) | **.68** | - | - |
| NEO-FFI 4 | - | - | **.66** |
| NEO-FFI 14 (R) | **.79** | -.10 | - |
| NEO-FFI 34 | **.34** | -.12 | .22 |
| NEO-FFI 49 | -.10 | - | **.70** |
| NEO-FFI 9 (R) | **.38** | - | - |
| NEO-FFI 54 (R) | - | **.34** | .14 |
| NEO-FFI 44 (R) | **.27** | .24 | - |
| NEO-FFI 59 (R) | **.55** | - | - |
| Note. NEO-FFI = NEO-Five Factor Inventory; Strongest loading for each item is bolded; loadings with an absolute value less than .10 were removed; (R) = reverse-coded. | | | |
|  |  |  |  |
|  |  |  |  |

Table S4

| Correlations between personality aspects and psychopathology indices | | | | | |  |  |  |  |  |  |
| --- | --- | --- | --- | --- | --- | --- | --- | --- | --- | --- | --- |
|  | 1 | 2 | 3 | 4 | 5 | | 6 | 7 | 8 | 9 | 10 |
| 1. N1 Volatility | - |  |  |  |  | |  |  |  |  |  |
| 2. N2 Withdrawal | .40* | - |  |  |  | |  |  |  |  |  |
| 3. E1 Assertiveness | -.04 | -.30* | - |  |  | |  |  |  |  |  |
| 4. E2 Enthusiasm | -.19* | -.31* | .50* | - |  | |  |  |  |  |  |
| 5. O1 Aesthetic Openness | -.05 | .09* | -.02 | .10* | - | |  |  |  |  |  |
| 6. O2 Intellect | -.04 | -.05 | .12* | .08 | .52* | | - |  |  |  |  |
| 7. A1 Selfish | .35* | .26* | -.07 | -.40* | -.07 | | .04 | - |  |  |  |
| 8. A2 Guarded | .46* | .29* | -.03 | -.37* | -.18* | | .00 | .75* | - |  |  |
| 9. A3 Inconsiderate | .25* | .22* | -.14* | -.38* | -.16* | | -.13* | .72* | .51* | - |  |
| 10. D1 Indolence | .09 | .39* | -.35* | -.19* | .17* | | .05 | .22* | .09 | .26* | - |
| 11. D2 Disorderliness | .13* | .39* | -.16* | -.13* | .11* | | .04 | .26* | .18* | .32* | .62* |
| Note. A = Antagonism / reversed Agreeableness; N = Neuroticism; E = Extraversion; O = Openness to new experience; D = Disinhibition / reversed Conscientiousness; **p* < .005 | | | | | | | | | | | |

Table S5

| Correlations between fMRI theory of mind processing, personality, and psychopathology | | | | | |
| --- | --- | --- | --- | --- | --- |
|  | Dorsomedial  Prefrontal | Temporoparietal  Junction | | Temporal Pole | |
| Hypothesis-driven variables |  |  |  | |  |
| A1 Selfish | -.02 | -.02 | -.02 | | |
| A2 Guarded | -.04 | .00 | .01 | | |
| A3 Inconsiderate | .02 | -.03 | -.03 | | |
| Exploratory variables |  |  |  | | |
| Manipulation item (NEO Item 59) | -.02 | -.02 | .01 | | |
| N1 Volatility | .00 | -.01 | -.03 | | |
| N2 Withdrawal | .00 | -.01 | -.01 | | |
| E1 Assertiveness | -.06 | -.02 | -.02 | | |
| E2 Enthusiasm | -.02 | .01 | .04 | | |
| O1 Aesthetic Openness | -.01 | -.01 | .03 | | |
| O2 Intellect | .02 | -.04 | .05 | | |
| D1 Indolence | .00 | -.03 | .00 | | |
| D2 Disorderliness | .03 | -.03 | .03 | | |
| Note. A = Antagonism / reversed Agreeableness; N = Neuroticism; E = Extraversion; O = Openness to new experience; D = Disinhibition / reversed Conscientiousness; Internalizing and Externalizing represent psychopathology composites of relevant ASR subscales; **p* < .005 | | | | | |

Table S6

| Correlations between Psychophysiological Interaction coefficients and Antagonism aspects and Externalizing | | | | | | | |
| --- | --- | --- | --- | --- | --- | --- | --- |
|  | Seed: Dorsomedial Prefrontal | | Seed: Temporoparietal  Junction | | Seed: Temporal Pole | | |
|  | Temporoparietal Junction | Temporal Pole | Dorsomedial Prefrontal | Temporal Pole | Dorsomedial Prefrontal | | Temporoparietal  Junction |
| Hypothesis-driven variables |  |  |  |  |  |  | |
| A1 Selfish | .00 | .01 | .00 | .02 | .03 | | .01 |
| A2 Guarded | .03 | .03 | .03 | .07 | .03 | | .04 |
| A3 Inconsiderate | .02 | -.01 | .02 | .00 | .04 | | .01 |
| Exploratory variables |  |  |  |  |  | |  |
| Manipulation item (NEO Item 59) | .03 | .05 | .00 | .02 | .05 | | .01 |
| N1 Volatility | .05 | .08 | .03 | .07 | .06 | | .04 |
| N2 Withdrawal | .01 | .03 | .02 | .01 | .01 | | .05 |
| E1 Assertiveness | .04 | .04 | .00 | .01 | .00 | | -.05 |
| E2 Enthusiasm | .02 | .01 | -.04 | -.04 | -.06 | | -.06 |
| O1 Aesthetic Openness | -.04 | -.02 | -.02 | -.01 | -.01 | | .05 |
| O2 Intellect | .02 | .01 | -.03 | -.04 | -.04 | | .00 |
| D1 Indolence | -.07 | -.05 | -.04 | -.02 | .00 | | .03 |
| D2 Disorderliness | -.02 | -.01 | -.01 | -.02 | -.01 | | .05 |
| Note. A = Antagonism / reversed Agreeableness; Externalizing represents psychopathology composite of relevant ASR subscales; *p < .005 | | | | | | | |

Table S7

| Exploratory factor analysis of NEO-FFI and ASR Item loadings | | | | | | |
| --- | --- | --- | --- | --- | --- | --- |
| Subscales / Facets | Item | NA | AE | E | D | O |
| N6 Vulnerability | NEO FFI 11 | **.62** | -.15 | .11 | -.20 | -.16 |
| ASR Anxious/Depressed | ASRVIII.ASR_047 | **.57** | -.21 | -.18 | -.15 | - |
| ASR Anxious/Depressed | ASRVIII.ASR_013 | **.56** | - | - | -.10 | - |
| ASR Aggression | ASRVIII.ASR_055 | **.56** | .10 | - | - | .11 |
| N3 Depression | NEO FFI 26 | **.55** | - | -.14 | -.24 | - |
| ASR Anxious/Depressed | ASRVIII.ASR_014 | **.55** | -.12 | .11 | - | - |
| ASR Aggression | ASRVIII.ASR_116 | **.55** | .10 | - | - | -.11 |
| ASR Anxious/Depressed | ASRVIII.ASR_052 | **.54** | - | - | - | - |
| N1 Anxiety | NEO FFI 31 (R) | **.54** | -.16 | - | -.15 | - |
| ASR Somatic | ASRVIII.ASR_056h | **.53** | - | - | - | - |
| ASR Somatic | ASRVIII.ASR_056c | **.53** | - | - | - | - |
| ASR Anxious/Depressed | ASRVIII.ASR_022 | **.51** | - | - | -.10 | - |
| ASR Somatic | ASRVIII.ASR_056f | **.50** | - | - | - | - |
| ASR Aggression | ASRVIII.ASR_087 | **.50** | .23 | - | - | - |
| ASR Somatic | ASRVIII.ASR_054 | **.49** | - | - | - | - |
| N1 Anxiety | NEO FFI 21 | **.49** | - | - | -.17 | -.13 |
| N3 Depression | NEO FFI 41 | **.49** | - | - | -.30 | -.13 |
| ASR Anxious/Depressed | ASRVIII.ASR_107 | **.48** | - | -.10 | - | - |
| ASR Anxious/Depressed | ASRVIII.ASR_071 | **.47** | -.13 | -.19 | - | - |
| ASR Somatic | ASRVIII.ASR_051 | **.47** | - | - | - | - |
| N3 Depression | NEO FFI 16 (R) | **.45** | - | -.16 | -.15 | - |
| ASR Anxious/Depressed | ASRVIII.ASR_033 | **.43** | - | -.17 | - | - |
| N4 Self-consciousness | NEO FFI 06 | **.43** | -.15 | - | -.19 | -.15 |
| ASR Somatic | ASRVIII.ASR_056b | **.41** | - | - | .13 | - |
| N1 Anxiety | NEO FFI 01 (R) | **.41** | -.24 | - | - | -.14 |
| N3 Depression | NEO FFI 46 (R) | **.40** | - | -.10 | - | - |
| ASR Anxious/Depressed | ASRVIII.ASR_031 | **.40** | - | - | - | - |
| ASR Anxious/Depressed | ASRVIII.ASR_113 | **.40** | - | - | -.11 | .11 |
| N6 Vulnerability | NEO FFI 51 | **.40** | - | - | -.38 | -.15 |
| N4 Self-consciousness | NEO FFI 56 | **.38** | - | -.12 | -.24 | - |
| ASR Somatic | ASRVIII.ASR_100 | **.38** | - | - | - | - |
| ASR Somatic | ASRVIII.ASR_056i | **.36** | .13 | - | - | - |
| ASR Aggression | ASRVIII.ASR_068 | **.35** | .23 | .14 | - | -.10 |
| ASR Anxious/Depressed | ASRVIII.ASR_091 | **.35** | - | - | - | .14 |
| ASR Aggression | ASRVIII.ASR_118 | **.34** | .22 | .14 | - | -.18 |
| ASR Somatic | ASRVIII.ASR_056a | **.34** | - | - | - | - |
| ASR Withdrawn | ASRVIII.ASR_060 | **.33** | .12 | -.21 | - | - |
| ASR Somatic | ASRVIII.ASR_056g | **.32** | - | - | - | - |
| ASR Anxious/Depressed | ASRVIII.ASR_034 | **.31** | .29 | -.11 | .15 | - |
| ASR Aggression | ASRVIII.ASR_005 | **.29** | - | - | -.11 | - |
| ASR Somatic | ASRVIII.ASR_056e | **.28** | - | - | .10 | - |
| ASR Withdrawn | ASRVIII.ASR_030 | **.25** | - | -.24 | - | - |
| ASR Rule Breaking | ASRVIII.ASR_117 | **.25** | .24 | .10 | -.10 | - |
| ASR Rule Breaking | ASRVIII.ASR_114 | **.24** | .19 | - | -.10 | .11 |
| ASR Aggression | ASRVIII.ASR_028 | **.23** | .21 | - | .12 | - |
| ASR Somatic | ASRVIII.ASR_056d | **.20** | - | - | - | - |
| ASR Withdrawn | ASRVIII.ASR_065 | **.20** | .15 | -.20 | - | - |
| A3 Altruism | NEO FFI 14 (R) | .15 | **-.53** | .13 | .20 | .11 |
| A2 Straightforwardness | NEO FFI 59 (R) | .16 | **-.51** | - | .17 | - |
| ASR Intrusive | ASRVIII.ASR_074 | - | **.48** | .35 | - | - |
| A4 Compliance | NEO FFI 54 (R) | - | **-.48** | - | -.13 | .10 |
| A4 Compliance | NEO FFI 09 (R) | - | **-.47** | - | - | .21 |
| ASR Aggression | ASRVIII.ASR_016 | .14 | **.45** | - | - | - |
| ASR Rule Breaking | ASRVIII.ASR_023 | - | **.44** | - | -.18 | .16 |
| ASR Intrusive | ASRVIII.ASR_094 | - | **.42** | .15 | - | - |
| ASR Rule Breaking | ASRVIII.ASR_043 | - | **.41** | - | -.11 | - |
| ASR Aggression | ASRVIII.ASR_095 | .24 | **.41** | - | .13 | -.16 |
| ASR Rule Breaking | ASRVIII.ASR_076 | - | **.40** | .10 | -.18 | .12 |
| ASR Rule Breaking | ASRVIII.ASR_039 | .12 | **.40** | - | - | - |
| E5 Excitement-seeking | NEO FFI 22 | -.12 | **.39** | .40 | - | - |
| ASR Rule Breaking | ASRVIII.ASR_092 | - | **.39** | - | - | .21 |
| ASR Rule Breaking | ASRVIII.ASR_041 | .16 | **.38** | .18 | - | - |
| ASR Aggression | ASRVIII.ASR_037 | .26 | **.36** | - | .22 | - |
| A6 Tendermindedness | NEO FFI 44 (R) | - | **-.36** | - | - | .23 |
| ASR Withdrawn | ASRVIII.ASR_025 | .20 | **.35** | -.13 | - | - |
| A1 Trust | NEO FFI 29 (R) | -.14 | **-.35** | .14 | -.13 | .21 |
| A4 Compliance | NEO FFI 19 | .21 | **-.35** | - | - | .14 |
| ASR Rule Breaking | ASRVIII.ASR_090 | - | **.33** | .13 | -.13 | .12 |
| ASR Intrusive | ASRVIII.ASR_007 | - | **.33** | .21 | -.16 | - |
| ASR Intrusive | ASRVIII.ASR_019 | - | **.33** | .29 | -.14 | - |
| A1 Trust | NEO FFI 24 (R) | - | **-.33** | .31 | - | .16 |
| ASR Rule Breaking | ASRVIII.ASR_026 | - | **.32** | - | - | - |
| ASR Rule Breaking | ASRVIII.ASR_006 | - | **.32** | - | - | .28 |
| ASR Aggression | ASRVIII.ASR_086 | .29 | **.31** | - | - | -.11 |
| ASR Aggression | ASRVIII.ASR_097 | - | **.30** | - | - | - |
| ASR Aggression | ASRVIII.ASR_081 | .25 | **.29** | - | - | .12 |
| ASR Rule Breaking | ASRVIII.ASR_082 | - | **.28** | - | - | .18 |
| N2 Angry Hostility | NEO FFI 36 | .30 | **.27** | - | - | -.19 |
| ASR Aggression | ASRVIII.ASR_057 | - | **.25** | - | .16 | - |
| ASR Rule Breaking | ASRVIII.ASR_020 | .20 | **.25** | - | - | - |
| ASR Rule Breaking | ASRVIII.ASR_122 | .20 | **.25** | - | - | .10 |
| E6 Positive Emotions | NEO FFI 37 | - | - | **.65** | .16 | .10 |
| E1 Warmth | NEO FFI 17 | - | - | **.61** | - | - |
| E2 Gregariousness | NEO FFI 02 | - | - | **.56** | - | - |
| E2 Gregariousness | NEO FFI 27 (R) | - | -.17 | **.49** | -.12 | - |
| E6 Positive Emotions | NEO FFI 42 (R) | - | -.10 | **.49** | .12 | .19 |
| ASR Withdrawn | ASRVIII.ASR_042 | .13 | .13 | **-.49** | - | .18 |
| ASR Intrusive | ASRVIII.ASR_104 | .11 | .34 | **.48** | -.11 | - |
| ASR Intrusive | ASRVIII.ASR_093 | .24 | .22 | **.47** | - | - |
| E6 Positive Emotions | NEO FFI 07 | - | - | **.44** | - | .11 |
| A3 Altruism | NEO FFI 39 (R) | .26 | -.50 | **.41** | - | - |
| E3 Assertiveness | NEO FFI 57 (R) | -.17 | - | **.36** | .11 | -.10 |
| ASR Withdrawn | ASRVIII.ASR_067 | .28 | .10 | **-.35** | - | .11 |
| E4 Activity | NEO FFI 32 | -.13 | .28 | **.33** | .17 | .14 |
| E4 Activity | NEO FFI 47 | - | .15 | **.32** | .12 | - |
| ASR Withdrawn | ASRVIII.ASR_069 | .18 | .19 | **-.31** | - | .13 |
| ASR Withdrawn | ASRVIII.ASR_111 | .23 | .16 | **-.28** | - | .14 |
| E6 Positive Emotions | NEO FFI 12 (R) | - | - | **.27** | - | .23 |
| A3 Altruism | NEO FFI 34 | - | -.21 | **.27** | .15 | .17 |
| C5 Self-discipline | NEO FFI 50 | - | - | - | **.65** | - |
| C4 Achievement Striving | NEO FFI 35 | - | - | .17 | **.57** | - |
| C4 Achievement Striving | NEO FFI 25 | - | - | - | **.56** | - |
| C3 Dutifulness | NEO FFI 40 | - | - | - | **.54** | - |
| C4 Achievement Striving | NEO FFI 60 | - | - | .13 | **.54** | .10 |
| C5 Self-discipline | NEO FFI 10 | - | - | - | **.54** | -.10 |
| C3 Dutifulness | NEO FFI 45 (R) | -.15 | -.15 | - | **.52** | -.13 |
| C2 Order | NEO FFI 55 (R) | -.16 | -.10 | -.11 | **.47** | - |
| C5 Self-discipline | NEO FFI 30 (R) | - | -.12 | - | **.46** | -.14 |
| C2 Order | NEO FFI 05 | - | -.12 | - | **.43** | - |
| O4 Actions | NEO FFI 08 (R) | - | - | - | **-.38** | .16 |
| C3 Dutifulness | NEO FFI 20 | .21 | -.26 | - | **.36** | .14 |
| O1 Fantasy | NEO FFI 03 (R) | - | - | - | **-.33** | .28 |
| E4 Activity | NEO FFI 52 | -.12 | .10 | .28 | **.29** | - |
| C2 Order | NEO FFI 15 (R) | - | - | -.14 | **.27** | - |
| O2 Aesthetics | NEO FFI 13 | .12 | - | - | - | **.59** |
| O5 Ideas | NEO FFI 48 (R) | - | - | - | - | **.57** |
| O5 Ideas | NEO FFI 58 | -.16 | .20 | - | - | **.57** |
| O2 Aesthetics | NEO FFI 43 | .20 | - | - | - | **.54** |
| O5 Ideas | NEO FFI 53 | -.13 | .13 | - | .10 | **.50** |
| O2 Aesthetics | NEO FFI 23 (R) | .13 | -.10 | - | - | **.49** |
| O4 Actions | NEO FFI 28 | - | .10 | .15 | - | **.37** |
| A3 Altruism | NEO FFI 49 | .21 | -.28 | .13 | .28 | **.35** |
| O6 Values | NEO FFI 18 (R) | - | - | - | - | **.34** |
| O3 Feelings | NEO FFI 33 (R) | - | -.20 | - | - | **.32** |
| A3 Altruism | NEO FFI 04 | .13 | -.28 | .15 | .22 | **.28** |
| O6 Values | NEO FFI 38 (R) | -.15 | - | -.20 | -.14 | **.27** |
| Correlations between factors |  |  |  |  |  |  |
| Antagonistic Externalizing |  | .42 | - |  |  |  |
| Extraversion |  | -.27 | -.15 | - |  |  |
| Disinhibition |  | .24 | .13 | -.21 | - |  |
| Openness |  | .07 | .09 | .12 | -.02 | - |
| Note. NA = Negative Affectivity; AE = Antagonistic Externalizing; E =  Extraversion; D = Disinhibition / Reversed Conscientiousness; O = Openness; N =  Neuroticism; A = Agreeableness. | | | | | | |

Table S8

| Correlations between fMRI ToM processing and combined ASR and NEO-FFI factors | | | | |
| --- | --- | --- | --- | --- |
|  | Dorsomedial  Prefrontal | Temporoparietal  Junction | | Temporal Pole |
| Negative Affectivity | .00 | .00 | -.01 | |
| Antagonistic Externalizing | .01 | -.01 | .01 | |
| Extraversion | -.02 | -.01 | .03 | |
| Disinhibition | .03 | -.03 | .01 | |
| Openness | .00 | -.02 | .07 | |
| Note. Disinhibition = reversed Conscientiousness; **p* < .005 | | | | |

Table S9

| Correlations between Psychophysiological Interaction coefficients and combined ASR and NEO-FFI factors | | | | | | |
| --- | --- | --- | --- | --- | --- | --- |
|  | Seed: Dorsomedial Prefrontal | | Seed: Temporoparietal  Junction | | Seed: Temporal Pole | |
|  | Temporoparietal Junction | Temporal Pole | Dorsomedial Prefrontal | Temporal Pole | Dorsomedial Prefrontal | Temporoparietal  Junction |
| Negative Affectivity | .01 | .03 | -.01 | .01 | .03 | .05 |
| Antagonistic Externalizing | .06 | .09* | .00 | .06 | .06 | .03 |
| Extraversion | .02 | .03 | -.02 | -.02 | -.03 | -.03 |
| Disinhibition | -.05 | -.05 | -.04 | -.03 | -.02 | .04 |
| Openness | .01 | -.01 | -.03 | -.03 | -.04 | .03 |
| Note. Disinhibition = reversed Conscientiousness; *p < .005 | | | | | | |
